# The complete chloroplast genome of *Castanea mollissima* ‘Chuizhili’

**DOI:** 10.1080/23802359.2021.1903360

**Published:** 2021-03-26

**Authors:** Shijie Zhang, Cancan Zhu, Xiaoqian Bai, Mimi Li, Yu Chen, Yuqiang Zhao

**Affiliations:** Institute of Botany, Jiangsu Province and Chinese Academy of Sciences, Nanjing, China

**Keywords:** *Castanea mollissima*, complete chloroplast genome, phylogenetic analysis, weeping chestnut

## Abstract

The *Castanea mollissima* ‘Chuizhili’ is an important variety of *C. mollissima* in breeding for dwarf chestnut and ornamental trees due to the weeping characteristic in China. In this study, the complete chloroplast genome of *C. mollissima* ‘Chuizhili’ was identified and sequenced by using Illumina sequencing data. The genome size is 160,799 bp, with a large single-copy (LSC, 90,430 bp) region, a small single-copy (SSC, 18,997 bp) region, and separated by a pair of 25,686 bp inverted repeat (IR) regions. A total of 130 genes are successfully annotated, including 83 protein-coding genes, 37 tRNA genes, 8 rRNA genes, and 2 pseudo-genes. The phylogenetic relationships revealed that *C. mollissima* ‘Chuizhili’ is closely related to *Castanea henryi* in Fagaceae.

The diversity of Chinese Chestnut (*Castanea mollissima* Bl.) includes many genetic traits of agronomic significance such as dwarf or weeping habit, early bearing, and high productivity, a wide range of ripening times as well as colored leaves, etc. (Craddock and Bassi 1999). *Castanea mollissima* cultivar ‘Chuizhili’ is a variety of *C. mollissima* and is called the weeping chestnut due to the characteristics of drooping branches in China (Sun et al. 2013). It is mainly grown in the areas of Shandong province and the eastern regions of China (Sun et al. 2013). *Castanea mollissima* ‘Chuizhili’ is cultivated as a good variety for early fruit and high yield in China (Shen 2015). Besides, *C. mollissima* ‘Chuizhili’ is usually used in landscape gardens due to the trunk growing in twists and weeping characteristics (Shen 2015). Weeping chestnut is excellent germplasm with both ornamental and breeding. Although this variety is valuable, existing genomic resources and genetic studies are limited for weeping chestnuts. In this study, we characterized the complete chloroplast genome sequence of *C. mollissima* ‘Chuizhili’ which has weeping characteristics.

The fresh leaves of *C. mollissima* ‘Chuizhili’ were sampled from the Chestnut Germplasm Resource Nursery in Nanjing Botanical Garden Mem. Sun Yat-Sen (32°03′020.79′′N, 118°49′53.37′′E), Nanjing, Jiangsu province, China. The voucher specimen (No. 190823) is kept at the Herbarium of the Chestnut Germplasm Resources Repositories in Jiangsu Province, China. The total genomic DNA was extracted from fresh leaf tissue using the Plant Genomic DNA kit (Proteinssci Biotech Co., Ltd, Shanghai, China). The qualified DNA was interrupted randomly with 350 bp by the Covaris ultrasonic breaker for library construction. Sequencing libraries were generated using NEB Next^®^ Ultra DNA Library Prep Kit for Illumina^®^ (NEB, USA) following the manufacturer’s recommendations. The whole-genome sequencing was conducted on the Illumina Hiseq 4000 platform (Illumina, San Diego, CA, USA) with paired-end reads (150 bp) by Novogene, Beijing, China. The complete chloroplast genome of *C. mollissima* (GeneBank accession: HQ336406.1) as the reference genomes used for assembly and annotation. The high-quality reads were assembled with Novoplasty version 2.7.2 (Dierckxsens et al. 2017). Annotation of the complete chloroplast genome was performed by GeSeq (Tillich et al. 2017) and adjusted by manual in Geneious 11.1.5 (https://www.geneious.com/). The annotated complete chloroplast genome of *C. mollissima* ‘Chuizhili’ has been deposited into Genbank (accession number MW322901).

The complete chloroplast genome of *C. mollissima* ‘Chuizhili’ was 160,799 bp in length and contained two inverted repeats (IRa and IRb) regions of 25,686 bp each, the large single-copy (LSC) region and small single-copy (SSC) region of 90,430 and 18,997 bp, respectively. The genome contained 130 genes, including 83 protein-coding genes, 37 tRNA genes, 8 rRNA genes, and 2 pseudo-genes. The GC content of the complete chloroplast genome was 36.8%. The GC content in LSC, SSC, and IR regions were 34.6, 30.8, and 42.8%, respectively.

To reveal the taxonomic status of *C. mollissima* ‘Chuizhili,’ phylogenetic analysis was performed based on 20 complete chloroplast genomes of Fagaceae and six taxa (*Carya illinoinensis*, *Corylus fargesii*, *Corylus chinensis*, *Juglans regia*, *Malus prunifolia*, *Populus tomentosa*) as outgroups. The chloroplast genomes were downloaded from NCBI GenBank. All chloroplast genomes were aligned using the MAFFT version 7.409 (Katoh and Standley 2013). The phylogenetic inference was generated based on maximum likelihood (ML) analysis with the GTR + G model in RAxML (Stamatakis 2014). 1000 bootstrap replicates were computed. The phylogenetic tree showed that *C. mollissima* ‘Chuizhili’ was most closely related to *Castanea henryi* in Fagaceae with bootstrap support values of 100% (Figure 1).

**Figure 1.**
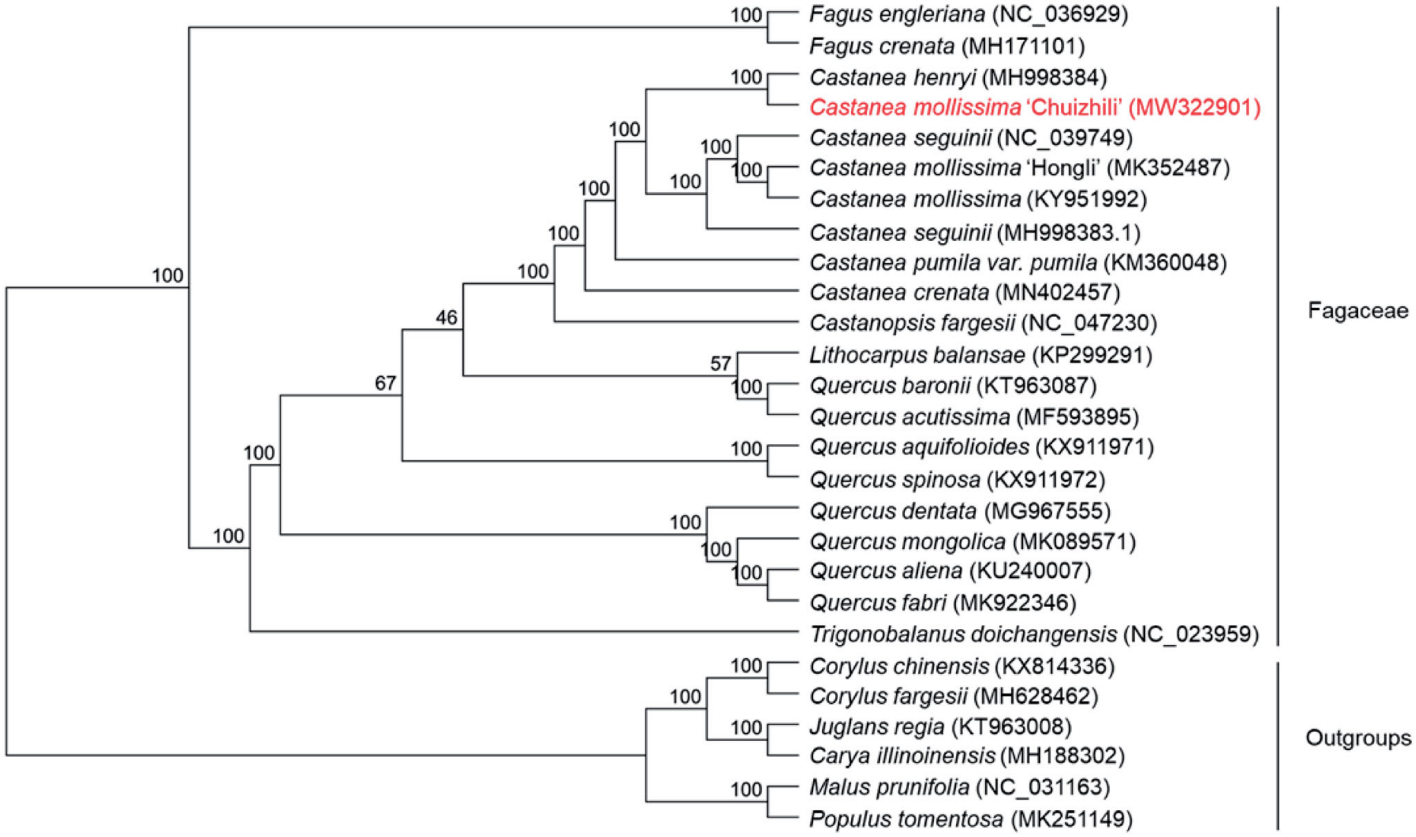
Phylogenetic analysis was performed by maximum-likelihood (ML) method based on 20 complete chloroplast genomes of Fagaceae and six taxa (*Carya illinoinensis*, *Corylus fargesii*, *Corylus chinensis*, *Juglans regia*, *Malus prunifolia*, *Populus tomentosa*) as outgroups. 1000 bootstrap replicates were computed. Numbers above the branches represent the bootstrap support values (%).

## Data Availability

The data that support the findings of this study are openly available in GenBank (https://www.ncbi.nlm.nih.gov) with the accession number is MW322901.
